# Endoscopic Management of Ingested Narcotic Substances: A Case Report and Literature Review

**DOI:** 10.7759/cureus.25058

**Published:** 2022-05-16

**Authors:** Anmol Mittal, Kamal Amer, Anjella Manoharan, Julien Hohenleitner, Richard Arrigo

**Affiliations:** 1 Internal Medicine, Rutgers New Jersey Medical School, Newark, USA; 2 Gastroenterology and Hepatology, Rutgers New Jersey Medical School, Newark, USA; 3 Internal Medicine-Pediatrics, Rutgers New Jersey Medical School, Newark, USA; 4 Gastroenterology and Hepatology, Hunterdon Gastroenterology Associates, Hunterdon, USA

**Keywords:** body packing, body stuffing, heroin ingestion, advance endoscopy, ingestion of toxic materials

## Abstract

Management of swallowed narcotics remains in contention despite the increased frequency of occurrence. International societies recommend conservative therapy with escalation to surgical interventions in cases where drug packets do not progress. However, multiple studies demonstrate a treatment benefit of endoscopic intervention. We report the case of a 27-year-old male who presented after ingesting heroin bundles and failed the 48-hour of conservative therapy. Repeat computed tomography scanning demonstrated no movement of the package. Endoscopic retrieval was successful, and the patient was discharged the same day. Endoscopic intervention in the removal of bagged narcotics should be considered in patients presenting after purposely ingesting narcotics as means of planned concealment.

## Introduction

Body packing is the pre-planned act of swallowing packets of illicit substances to conceal and transport them surreptitiously [1]. Body stuffing, in contrast, refers to the unplanned concealment of loosely wrapped drugs [2]. This growing narcotic epidemic is raising concerns as there are established diagnosis parameters but controversial treatment modalities [3]. Guidelines do not differentiate between body packing and body stuffing, though there are clear differences in the theoretical risk of perforation between the two. Multiple international guidelines including the European Society of Gastrointestinal Endoscopy and the American Society for Gastrointestinal Endoscopy recommend a conservative "watchful waiting" technique with aggressive bowel therapy if needed [4-6]. Most packages are wrapped in thin-membraned products such as cellophane, miniature Ziploc bags, wax paper, latex, or even aluminum foil and, therefore, are considered at high risk of rupture. If conservative therapy fails, more invasive treatment is usually warranted. Any patient with drug containers present for greater than 48 hours, those presenting with packet ruptures, or those with evidence of bowel impaction present are typically referred for surgical removal [7,8]. Surgery is considered a safe practice due to the ability to create a local gastrostomy and remove packets with minimal difficulty [9,10]; however, there are risks of missed bags due to lack of visualization and rupture from intestinal milking [11]. Endoscopic removal of the drugs has seen increasing rates of use in medical practice with successful outcomes, specifically when performed in a highly controlled setting [12,13]. Here, we present a case of a body-packing patient with two large quantity stacks of heroin baggies removed successfully with limited endoscopic manipulation.

This article was previously presented as an oral presentation at the Eighth Annual Digestive Diseases: New Advances Conference held in Philadelphia, Pennsylvania, United States, on September 17-18, 2021.

## Case presentation

**Figure 1:**
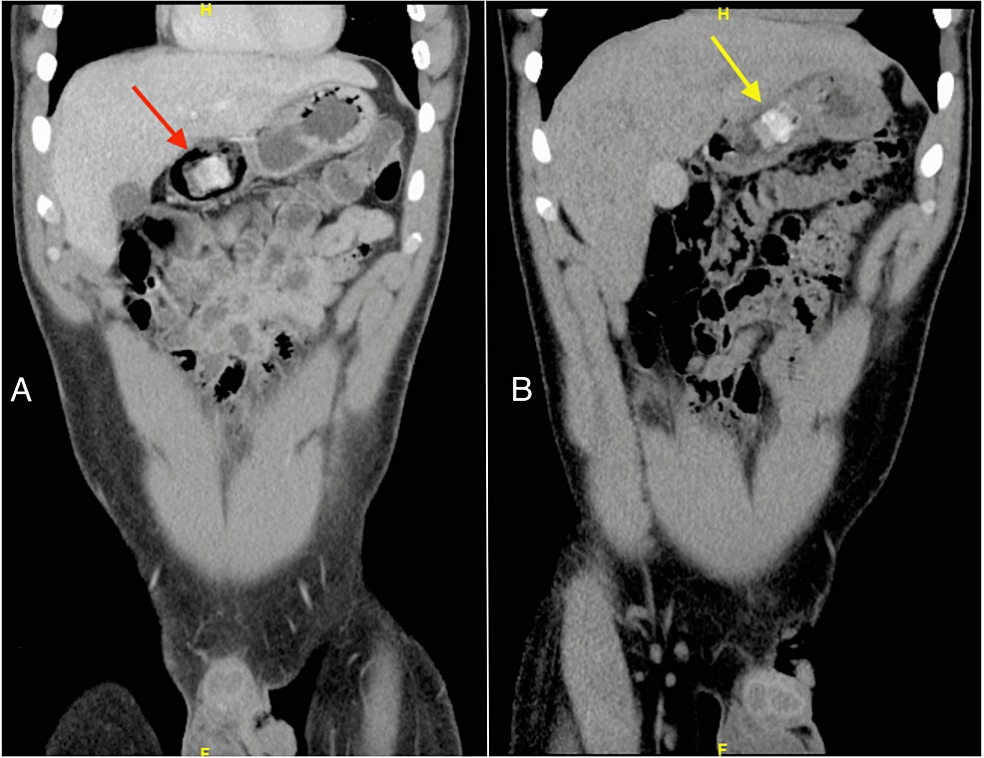
Computed tomography of the abdomen: (A) at admission, showing one of the packages in the gastric antrum (red arrow); (B) after 48 hours, showing the persistence of heroin bags in the gastric antrum with minimal movement (yellow arrow).

A 27-year-old male was brought into the emergency department in police custody due to abdominal pain with associated nausea and vomiting. Upon questioning, he admitted to swallowing thirty whole bags of heroin on the previous day, to hide them from the police. He reported that the bags were banded together by rubber bands in stacks of ten. He informed the team that after ingesting these stacks he had an episode of emesis leading to the prompt removal of one stack. However, the other two stacks remained in his system and to his knowledge had not passed through his digestive tract. On examination, his vital signs including heart rate, respiratory rate, and oxygen saturation were within normal limits. His laboratory examination demonstrated no abnormalities, and his urine toxicology screen was negative. An abdominal CT scan done at presentation demonstrated dense dependent material within the gastric antrum, thought to be the two stacks of heroin packets (Figure 1A).

**Figure 2:**
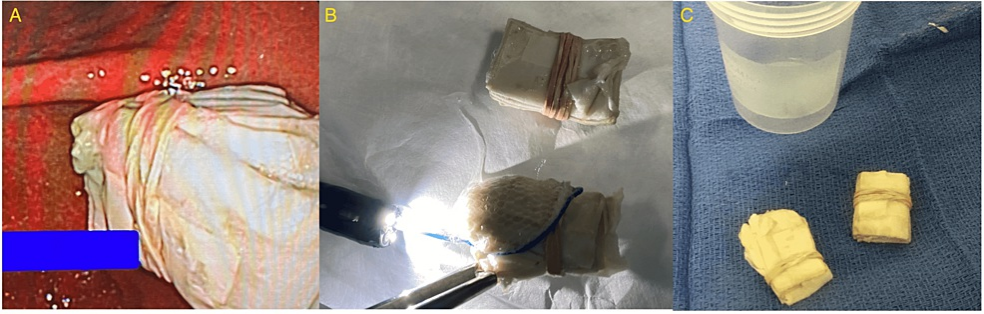
(A) Endoscopic view of the heroin bags inside gastric antrum; (B) and (C) Successful retrieval of two heroin bundles through snare.

Due to a lack of evidence suggesting bowel obstruction or packet rupture, the Gastroenterology team recommended conservative management. Poly-ethylene glycol (PEG) was utilized without success, and he was admitted for observation with a Narcan IV drip on standby due to the risk for severe central nervous system and respiratory depression. A repeat CT scan demonstrated similar findings of dense, dependent material in the gastric antrum (Figure 1B). After 72 hours of aggressive bowel preparation, the patient had not excreted the packets. On the morning of the fourth day, the patient became bradycardic. As a result, an esophagogastroduodenoscopy (EGD) extraction was attempted in the operating room (OR) with a naloxone drip, and the surgery team at the bedside, ready to change to an open exploratory laparotomy in the event of a bag rupture. Twenty packets divided into two stacks wrapped in rubber bands were extracted via snare (Figure 2). The patient tolerated the procedure without packet rupture or acute decompensation. Visual examination of packets post retrieval demonstrated no deformities or loss of integrity in the packaging and the patient was able to be discharged the same day. 

## Discussion

With the incidence of body packing and stuffing rising globally, it is important to consider appropriate and safe management techniques for this condition [1,14]. Conservative management with oral laxatives has been shown to be an effective first-line therapy in treating these individuals [14]. The second-line therapy continues to remain controversial as the benefits of endoscopic treatment are weighed against theoretical adverse events of rupture. The competing treatment using surgical techniques demonstrates the same possibility of adverse events but also increases the chances of missing smaller bags that may not be visualized on imaging [15]. Though there are guidelines in place for the retrieval of these packages, there are several facets to this scenario that are not considered in these recommendations.

First, there should be a dichotomy factoring in if the ingestion was from packing or stuffing. It is established that patients who present after body stuffing are less likely to have a secured package inside their body leading to a higher acuity situation; however, those that present after body packing typically have well-packed drugs inside, which are safer to manipulate. While multiple studies have demonstrated the safety of endoscopic retrieval in body stuffers, this attribute to the presentation may be considered when deciding on therapy [12,15]. Another facet to consider alongside these differences is the time for presentation between these groups. Body stuffers, due to the unplanned ingestion, will likely present much earlier than planned drug packers, which means they will have less progression of the packet in the GI tract. Given that endoscopy is limited to the second part of the duodenum, the timing is important to consider EGD as a possibility [15]. Although there are risks associated with endoscopic removal of these illicit substances, especially heroin, the case described here demonstrates that with the proper precautions, endoscopy can be an effective means of decontamination. In the case of heroin intoxication, endoscopy can be considered in an OR with a naloxone drip and a general surgery team on standby in the case of an unsuccessful endoscopic procedure.

Finally, due to the decreased morbidity and mortality from endoscopic intervention, more patients may qualify for intervention compared to restrictions placed on patients with the high operating risk associated with their comorbidities and anesthetic use. This in combination with the decrease in hospital costs and length of stay for patients undergoing endoscopic makes using endoscopy an attractive option [16]. Our case contributes to other case studies demonstrating the superiority of endoscopic techniques in healthcare utilization including decreased length of stay, hospitalization costs, and provider expenses.

## Conclusions

Body stuffing and body packing have become a severe problem in the United States in recent years. Currently, the guidelines for treatment do not align with the most recent literature published. A distinction in treatment modality should be made for those presenting with body stuffing vs. body packing, given the differences in preparation of the packets and risk of rupture. It is also essential to evaluate the risks and benefits of endoscopic vs. surgical intervention, especially as the literature and our case demonstrate the safer outcomes of endoscopic intervention. We should prioritize endoscopic evaluation with careful planning for the treatment of patients presenting with toxic ingestions as they may lead to improved healthcare utilization and safer results.
